# Effective visualization of integrated knowledge and data to enable informed decisions in drug development and translational medicine

**DOI:** 10.1186/1479-5876-11-250

**Published:** 2013-10-08

**Authors:** Lena Brynne, Anders Bresell, Niclas Sjögren

**Affiliations:** 1 AstraZeneca, Södertälje SE-151 85, Sweden

**Keywords:** Data integration, Preclinical data, Clinical data, Informatics, Visualization, Decision-making, Translational medicine, Drug development, Knowledge management

## Abstract

Integrative understanding of preclinical and clinical data is imperative to enable informed decisions and reduce the attrition rate during drug development. The volume and variety of data generated during drug development have increased tremendously. A new information model and visualization tool was developed to effectively utilize all available data and current knowledge. The Knowledge Plot integrates preclinical, clinical, efficacy and safety data by adding two concepts: knowledge from the different disciplines and protein binding.

Internal and public available data were gathered and processed to allow flexible and interactive visualizations. The exposure was expressed as the unbound concentration of the compound and the treatment effect was normalized and scaled by including expert opinion on what a biologically meaningful treatment effect would be.

The Knowledge Plot has been applied both retrospectively and prospectively in project teams in a number of different therapeutic areas, resulting in closer collaboration between multiple disciplines discussing both preclinical and clinical data. The Plot allows head to head comparisons of compounds and was used to support Candidate Drug selections and differentiation from comparators and competitors, back translation of clinical data, understanding the predictability of preclinical models and assays, reviewing drift in primary endpoints over the years, and evaluate or benchmark compounds in due diligence comparing multiple attributes.

The Knowledge Plot concept allows flexible integration and visualization of relevant data for interpretation in order to enable scientific and informed decision-making in various stages of drug development. The concept can be used for communication, decision-making, knowledge management, and as a forward and back translational tool, that will result in an improved understanding of the competitive edge for a particular project or disease area portfolio. In addition, it also builds up a knowledge and translational continuum, which in turn will reduce the attrition rate and costs of clinical development by identifying poor candidates early.

## Background

Translational Medicine is the discipline focusing on improving drug discovery and development by bridging the gap between basic research, clinical development and clinical practice. The key is to identify and quantify biomarkers that characterize the efficacy and safety profiles at different stages of drug development. The goal is to build up the knowledge of a translational continuum from bed to bench and vice versa, e.g. forward and back translation [1]. The translation of the pharmacodynamic drug action between species is a fundamental process in order to confidently select drug candidates that will demonstrate the biological and translational hypothesis in clinical development, and therefore also reduce the attrition rate [2-4].

Within all development phases, it is imperative to visualize data to be able to explore and integrate biomarkers from preclinical and clinical studies for multiple compounds (for benchmarking, differentiation and to compare forerunners) side-by side for informed decisions. This requires aggregation of a large amount of data and a holistic scientific understanding of all biomarkers. The visualization and integration of different biomarkers and endpoints across a large variety of studies are tedious processes and requires input and collaboration between experts from multiple disciplines in the organization. A platform that addresses this requires seamless access of data from clinical trials and preclinical studies. Furthermore, it needs to encompass a framework for harmonizing the interpretation of different types of data, gathered from various species, patient populations and therapeutic areas. The platform should, both technically and organizationally, allow use and reuse of data retrieved from internal and external sources as well as outputs from pharmacokinetic and pharmacodynamic modeling and simulations (PKS™). It should also address both individual and aggregated data within and across projects. Export of data to other applications for visualization and integration is key to ensure flexibility.

A number of commercial informatics tools (e.g. D360 [5], TranSMART [6]) for translational research purposes allow searching, analyzing and sharing data from multiple sources, data types and scales, are currently available and enables export of data to other applications. TranSMART Knowledge Management Platform is a platform that combines a data repository with intuitive search capabilities and analysis tools. However, it is based on a gene-centric approach that supports hypothesis development from a phenotypic perspective.

Today, pre-defined integration and visualization of data to answer key questions are performed within the tight project timelines. The volume and variety of data generated during drug development have increased tremendously. The Napiergram [7] is widely used within Pharma to get an overview of preclinical and clinical data, by presenting exposure ranges of unbound drug concentrations across assay systems and biomarkers. There is a clear need to facilitate the tedious work of visualizing and managing data for forward and back translation of compounds, as well as compound comparison at each milestone.

This paper describes the Knowledge Plot, a new translational framework for effective and flexible integration of preclinical and clinical data using a project-centric approach. The Plot, utilize current knowledge and desired effect levels for each biomarker and opens up for a transparent discussion and holistic understanding. The principles of how to construct a Knowledge Plot will be outlined, as well as retrospective and prospective use cases, demonstration of the translational continuum, and how it is imperative to use real-time data integration.

## Method

**Figure 1:**
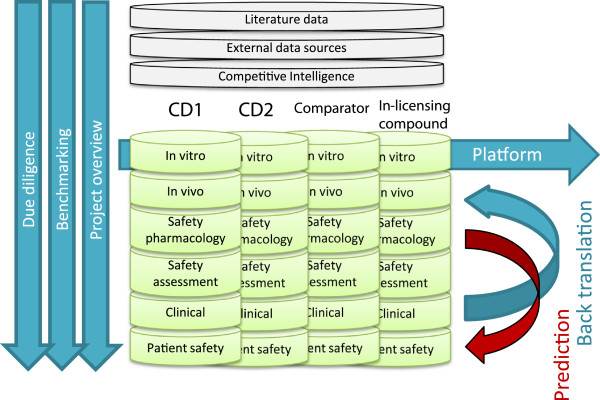
**Schematic overview of the knowledge plot data warehouse including the different sources.** Data can be integrated for different purposes, i.e. validation of platforms, overview of project data, decision-making, due diligence, benchmarking, and predictability of preclinical models and back translation of clinical data.

A target centric database was built and used as an intermediate data platform to manage the complex data in the translational space. Here, an Oracle database was used for data warehousing, Python for web-based data entering-GUI and TIBCO Spotfire for visual analytics. The Knowledge Plot approach is not technology dependent but interoperability within the system landscape should be taken into account. Ultimately, the technical solution is integrated with existing databases in-house as well as relevant external databases. Raw or processed data from the different studies were extracted from various data sources, reports and scientific articles. Data are typically aggregated for each dose or treatment group, but individual data can also be applied. Data sources used in this work are generically described in Figure 1.

### The Knowledge Plot

The basic Knowledge Plot has unbound concentration of the compound(s) of interest on the horizontal axis and a normalized and scaled summary measure of the treatment effect (placebo/vehicle and/or baseline controlled) for an endpoint on the vertical axis. The scaled treatment effect is expressed as an index and it is called the Treatment Effect Index (TEI), where a value of 100 means a Meaningful Effect and is defined as:

$$\text{Treatment Effect Index}\;\left(\mathrm{TEI}\right)=100\times \frac{\text{Treatment Effect}}{\text{Meaningful Effect}}$$

The Treatment Effect Index is calculated by normalizing the effect observed in the treatment group (Treatment Effect in the formula above) with the effect value that is considered to be relevant (biologically meaningful, i.e. Meaningful Effect above). The Treatment Effect is calculated for the actual endpoint taking account of the study design and potentially other facts important to get an unbiased estimate of the Treatment Effect (see examples of effect formulas that can be used to derive the Treatment Effect in Table 1). The Meaningful Effect is set according to thresholds pre-defined by key opinion leaders and/or internal expertise. Examples of Meaningful Effect criteria for clinical endings and biomarkers are: Target Product Profile and Target Product Claims criteria’s, clinical lab values above upper-limit of normal and per-cent of subjects reporting a certain type of severity of an adverse event. Examples for preclinical endpoints and biomarkers; pre-defined criteria’s in the Candidate Drug Target Profile or an effect level defined by a clinically validated reference drug.

**Table 1:** Examples of effect formulas

**Effect formula**	**Effect definition**	**Unit of reference value**
m	Unscaled (e.g. occupancy, %)	%
m - m_bl_	Change from baseline (raw)	Same unit as endpoint
m - m_ctrl_	Change from control (raw)	Same unit as endpoint
100*((m -m_bl_) / m_bl_)	Change from baseline (%)	%
100*((m - m_ctrl_) / (m_ctrl_)	Change from control (%)	%
100*(N_sbj,grp_/N_grp_ – N_sbj,ctrl_ /N_ctrl_)	Diff in % Event (percentage points)	% (percentage points)

M; mean response of endpoint at the corresponding exposure level.

m_bl_; mean response of endpoint at the baseline.

m_ctrl_; mean response of endpoint at the corresponding exposure level in the control group.

N_grp_; total number of subjects in the group.

N_sbj_,_grp_; number of subjects that experienced the event in the group.

N_ctrl_; number of subjects in the control group.

N_sbj,ctrl_; number of subjects that experienced the event in the control group.

The TEI should be transparent with a clear rationale, which is agreed within the project team or skill network or dictated by a governance body. TEI and the corresponding rationale are documented in the database in order to support a common understanding between experts of different endpoints. There are similarities between the previously published Clinical Utility Index [8-10] and the TEI presented in this paper. Hence, a comparison is outlined in the Discussion-section. Regarding the interpretation of TEI, a TEI value of 0 means no effect of treatment, a value between 0 and 100 means that the effect goes in the desired direction but the size of the effect has not reach the Meaningful Effect, a negative TEI value means that the effect goes in the undesired direction, and a value above 100 means that the effect is greater than the Meaningful Effect.

**Figure 2:**
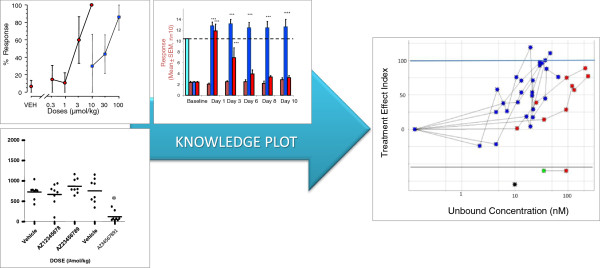
**Traditional visualizations compared to the knowledge plot.** The left part demonstrate a mixture of scatter, bar and line plots combined with tables. The right part illustrates the Knowledge Plot concentration-response profiles by using Treatment Effect Index on the vertical axis and the unbound concentration on the horizontal axis. Safety endpoints are colored by red and efficacy endpoints are blue to visualize the therapeutic window, which is the difference in exposure between the blue efficacy endpoint and the red safety endpoint on the horizontal 100-line. In addition, one point estimate representing a Ki value and a concentration range (no observed adverse effect level (NOAEL) and the lowest observed adverse effect level (LOAEL)) are shown in the lower part of the graph.

The left part of Figure 2, illustrates the traditional way of exploiting and visualizing data using different charts, e.g. bar, line and scatter. The right part of Figure 2 shows an example of how similar types of data are visualized in a Knowledge Plot. A point coordinate in the Knowledge Plot represents the mean unbound concentration (horizontal axis) for a certain dose group and its corresponding mean Treatment Effect Index (vertical axis) for a certain endpoint. Data points for a certain endpoint are connected with a line within each study. Note that different dose schedules, formulations, route of administration or duration should be separated as different treatment arms in the visualization for a certain endpoint. The majority of plots developed so far are based on group aggregates; however individual data can be plotted as well. The color of the point illustrates the type of endpoint (blue for efficacy, red for safety, yellow for target engagement, black for in vitro data etc.), whereas the shapes indicate the type of species. The bottom of the Knowledge Plot is similar to the Napiergram, where data are visualized as either point estimates (e.g. Ki, Kd, EC50, IC50) or exposure ranges e.g. no observed adverse effect level (NOAEL (green star)) and lowest observed adverse effect level (LOAEL (red star)) interval in a 3-month tox study, c.f. lower part of the right-hand plot in Figure 2. The safety margin or the therapeutic window [2] is expressed as the difference in exposure between efficacy and safety endpoints at the 100-line on the vertical axis. Outputs from pharmacokinetic and pharmacodynamics modeling and simulations are also entered to the database.

**Figure 3:**
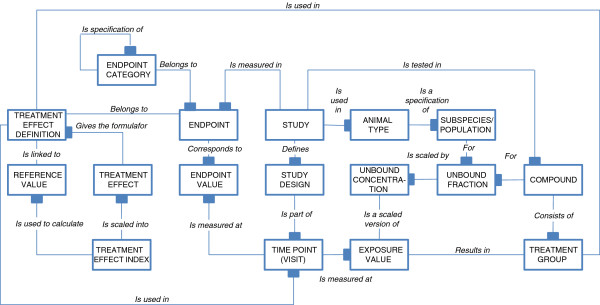
**Information model.** This conceptual information model illustrates all the key information entities (boxes) and relationships (lines) that are needed to fully utilize the Knowledge Plot. The relationships are of two types; one-to-one or one-to-many, where 'many’ is illustrated with filled anchor points. The relationship names are to be read from the entity with the anchor point.

In Figure 3, the information model describes the information objects that are needed to construct a Knowledge Plot in a basic situation, i.e. visualization of both safety and efficacy data from different studies with different doses and study designs. Other types of visualizations, such as plotting no adverse effect levels, Ki/Kd and EC50/IC50 values, pharmacokinetic-pharmacodynamic data could be constructed in a similar way as outlined in the basic situation described here.

In addition to data traditionally collected in a study (e.g. numerical measurements of exposure and endpoints), the Knowledge Plot requires the following additional information:

i) *Study meta data*.

Study information describing the study design, compounds, species/population, study code and other data outlined only in study documentation.

ii) *Controlled terminology*.

Terminology is generally consistent within a study, but across studies and across development programs the terminology is out of sync. Encoding all the various entities in a controlled fashion is especially important for species, endpoints and compounds.

iii) *Categorization of endpoints*.

Grouping and clustering of endpoints in a hierarchical manner allows comparison across compounds and species at different levels. In our approach the levels corresponds to the granularity of the key question.

At the top level: Is it a safety or efficacy endpoint?

Intermediate level: Which domains does the effect derive from? (Vital signs, biomarkers, adverse events etc.)

At the lowest level: What are the effects? (Increased blood pressure, occurrence of specific liver signals, number of subjects experience at least one occasion of dizziness etc.).

iv) *Specifications of the Treatment Effect Index.*

The specifications of how the Treatment Effect Index is calculated for each endpoint may vary between species, target and type of disease etc. These descriptions outline how the placebo/vehicle or baseline controlled response is derived (Treatment Effect Definition) and what a meaningful clinical or biological effect (Reference value) is on this scale. Table 1 contains a list of commonly used Treatment Effect Definitions and their associate brief explanations. The set of options for transforming endpoint measurements into a Treatment Effect, accounts for the different study designs.

v) *Unbound fraction (Plasma protein binding).*

The unbound fraction is specific for the species/strain and the compound. The factor allows comparison of the exposure levels across species by using the unbound concentrations rather than the total plasma concentration of the compound.

A data mart is built row-wise by combining maximum exposure and endpoint values by the treatment group (in an advanced mode the rows are further divided into time points and visits). Study meta information, unbound fraction (protein binding), treatment information, and scaling details are then added to each row. The unbound concentration is calculated by multiplying the free fraction by the mean total concentration in each treatment group. In this paper maximum plasma concentration (Cmax) and steady-state plasma concentration (Css) are used, but other measures reflecting the exposure (e.g. area under the curve (AUC)) can be used if that is more relevant for the actual disease area or compound properties. Output from pharmacokinetic-pharmacodynamic modeling is used when there is a lag-time between maximal exposure and effect (hysteresis) [2]. Pharmacokinetic data from satellite animals are used, when no other data are available. As an example, a list of variables that were collected to build a Knowledge Plot is given in Additional file 1: Table S1. For vocabularies, MeDRA [11] were used to describe adverse events, whilst internally developed vocabularies and data structures were used for the majority of other data types. The Treatment Effect is calculated for each endpoint and time point using the corresponding Treatment Effect Definition that corresponds to the study design. The Treatment Effect is then normalized into the Treatment Effect Index by using the reference value.

### Visualization

All available data from a project/target are exported into a data mart and loaded into the visualization tool. A tool that allows interactive visualizations is preferable as it gives possibility to switch between time, total and unbound concentration on the horizontal axis, as well as between raw data, the un-normalized treatment effect and Treatment Effect Index on the vertical axis. Thus, it is possible to visualize a certain endpoint using either raw or transformed data, or apply the treatment effect index when exploring the time or concentration relationships. The dose groups information, Treatment Effect Index rationale and other study meta information are stored in the database and easily accessible. Scatter plots is the recommended graph-type, where data points can be connected by a line for each endpoint and study. Furthermore, an interactive tool allows filtering out subsets of data to highlight selected endpoints, compounds and studies of interest. In addition, a tool that can color, shape, and size the points depending on their attributes are desired, so is also the possibility to draw trellis plots. Graphical templates can be optimized for each disease area or target of interest to enable instant visualization of data.

## Results

The Knowledge Plot uses two concepts, knowledge (c.f. TEI) and plasma protein binding. The concept of adding knowledge about each endpoint enables comparison of efficacy and safety data generated in the same species. By also adding protein binding for each species, comparisons between species, populations and subpopulations are possible and the number of ways to visualize and integrate data will be innumerable [2]. The Knowledge Plot can integrate studies performed on non-equivalent doses and transparently revile different receptor densities or pathophysiological mechanisms in different species, interference of safety pharmacology/toxicology endpoints with efficacy endpoints. Trellis plots are powerful when dissecting data in different ways and comparing compound profiles. Data from different compounds could either be plotted on top of each other in the same graph or side-by-side. The flexibility enables a relative comparison of information/knowledge within or between compounds by utilizing data from all development stages. Data are visualized on the same scale and uses the same unit, enabling eyeball inspection to compare drug profiles.

**Figure 4:**
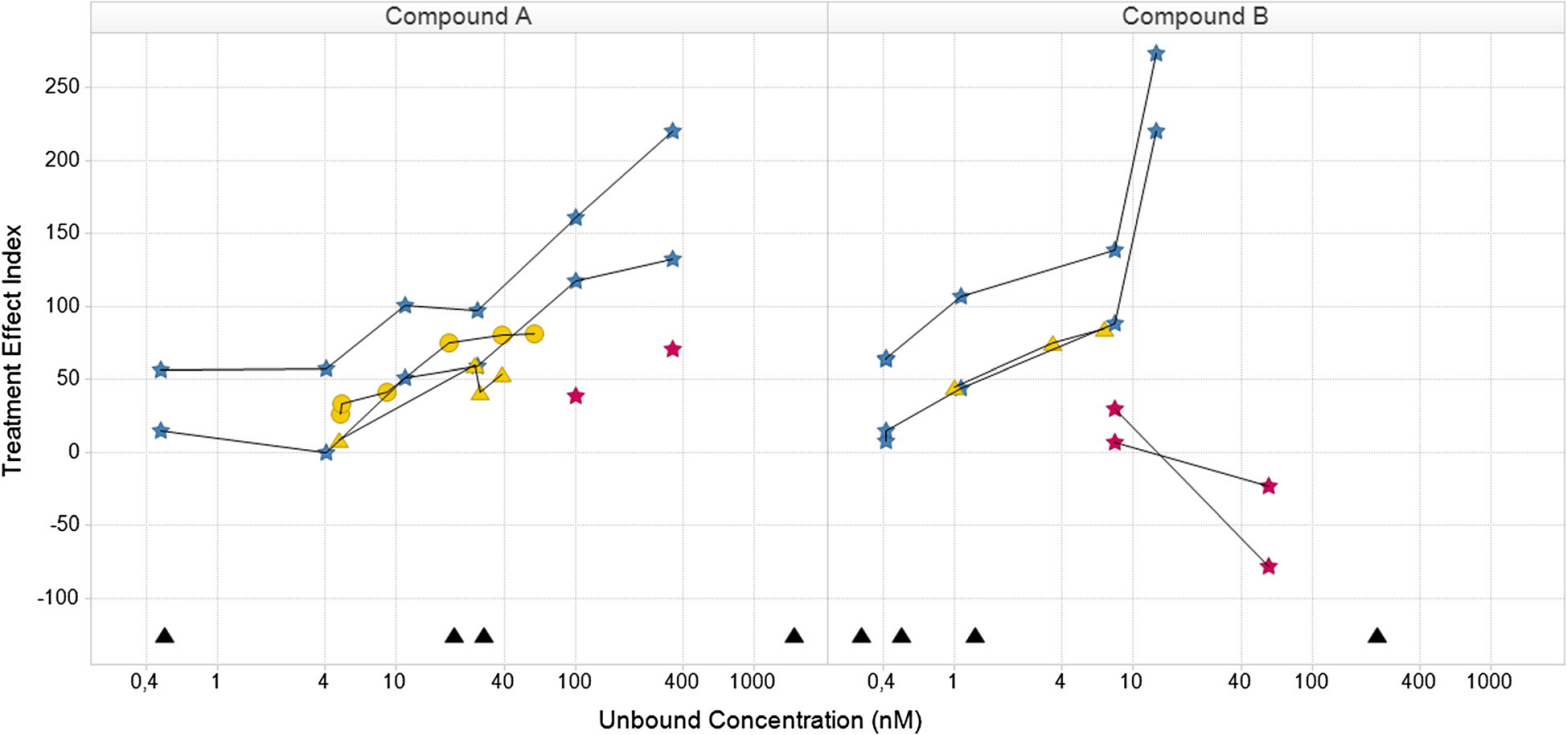
**Relationship in the knowledge plot between drug candidate and internal or external comparators with focus on preclinical efficacy and safety: comparison of efficacy and safety data for compound A and B.** Both compounds have similar efficacy profiles (blue). The safety profile for compound **A** is different from compound **B**, where compound **A** moves towards the Meaningful Effect i.e. is expected to demonstrate safety issues. The yellow curves describe target engagement for monkey (circles) and human (triangles) and will support the holistic understanding of the relationship between efficacy and safety vs level of target engagement (similar for rat (not shown), monkey and human). The black triangles in the lower part are human Ki-values for the different receptor subtypes derived from an in vitro assay. Note that there are two efficacy profiles for each compound, which corresponds to two different ways to assess efficacy. The concept of the Knowledge Plot is demonstrated here and all details of the biomarker, study information etc. are available in the data warehouse c.f. Appendix. Legends for species: rat (star), monkey (circle), human (triangle).

**Figure 5:**
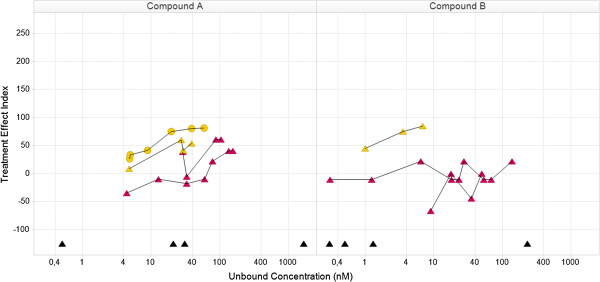
**Relationship in the knowledge plot between drug candidate and internal or external comparators with focus on adverse event in single and multiple dose studies: comparison of adverse event frequencies (red triangles) between compound A and B, including data from both single and multiple ascending dose studies.** The adverse event profiles are similar regardless of treatment duration time for the two compounds, respectively. However, there is a difference between compound **A** and **B**, where the former demonstrate a less favorable safety profile. By including target engagement c.f. Figure 4, there is a possibility to illustrate target related safety. Legends for species: rat (star), monkey (circle), human (triangle).

The Knowledge Plot was applied retrospectively and prospectively in selected projects within or across multiple therapeutic disease areas. In the retrospective analysis, preclinical and clinical key biomarkers and endpoints were selected i) for candidate drug selection, benchmarking and differentiation by utilizing all available knowledge on reference compounds and competitors, ii) to identify which data were used at each milestone for decision making, iii) to compare endpoints used in different subpopulations iv) back translation of clinical data to build translational continuum and understand the predictability of preclinical models and assays, v) evaluation of different compounds with different mechanism of actions aiming for the same patient population and vi) evaluate different dose schedules. The first example using the Knowledge Plot concept demonstrates the relative differences between two compounds (Compound A and B) by comparing their integrated profiles, supporting either candidate drug selection or benchmarking or differentiation with reference compound or competitor (Figures 4 and 5). The data included are: in vitro assays; target engagement; in vivo efficacy; safety pharmacology; and tolerability in early clinical studies. Each line represents the behavior of a specific endpoint with increasing exposure in different dose groups. The efficacy endpoint in the rat was calculated using two different methods (blue stars). Both methods gave similar profiles and reached the 100-line, e.g. the targeted or Meaningful Effect level. Target engagement in monkey and human (yellow circles and triangles) are similar and appears in both Figures 4 and 5. The difference between the two compounds is clearly demonstrated in the safety pharmacology endpoint (red stars in Figure 4), where compound A moves towards the 100-line, i.e. getting worse. Compound B moves in the opposite direction demonstrating getting better or no effect. The point estimates in the lower part of the curve are in vitro data (Kd, Ki, EC50 or IC50) and are there to recall the differences between the compounds. Their full in vitro profiles were visualized in a separate graph (unpublished data).

**Figure 6:**
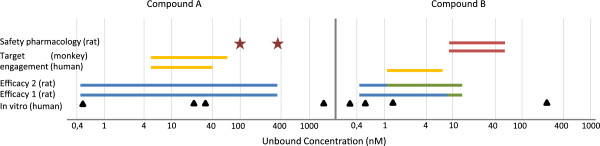
**Napiergram for compound A and B.** The unbound exposure ranges versus different preclinical and clinical endpoints or biomarkers using the same data as in Figure 4. The Knowledge Plot concept has been applied to Compound **B**, where the exposure range above the Meaningful Effect level is shown by change in color (from blue to green).

Figure 5 describes the relationship between mild adverse events and target engagement in two early clinical studies with different study designs, single and multiple dosing. There is one clear difference between the two compounds, where the adverse event endpoint for compound A moves towards the 100-line and not for compound B. This type of visualization can support dose selection, but that requires real-time access to data. The inclusion of target engagement data enables a better understanding of the adverse event profiles. The graphs also stimulate initial discussion of back translation of the adverse event endpoint to preclinical safety pharmacology data in the project team (red points in Figures 4 and 5). Additional data are needed to validate the predictability and face validity of the safety pharmacology endpoint. The Treatment Effect Index helps non-experts to get a holistic understanding of data. The project centric database enabled fast and flexible integration and visualization of data. Data were easy to reuse and instantaneously change the visualization for new critical questions or hypothesis. Traditional way of exploring data could be done by changing the concentration from free to total on the horizontal-axis and Treatment Effect Index to original observed mean values or reported Effect on the vertical-axis. If no protein binding are available, data can be visualized using total concentration only. The corresponding Napiergram for Compound A and B (Figure 4) are shown in Figure 6, where the Knowledge Plot concept is applied to Compound B. Note that the colored line changes from blue to green for the efficacy endpoint in the Napiergram at exposures that correspond to levels above the 100-line in the Knowledge Plot.

**Figure 7:**
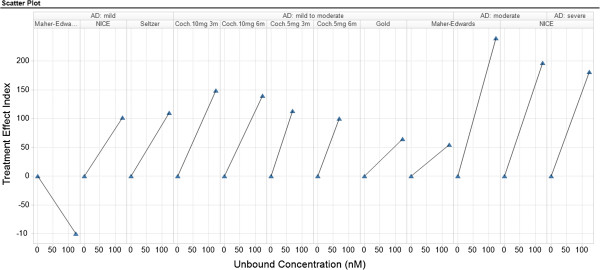
**Performance of ADAS-Cog test in different subpopulations of alzheimer’s disease visualized in the knowledge plot.** The Plot demonstrates that there are different responsiveness in ADAS-Cog in different subpopulations of Alzheimer’s Disease, treated with Donepezil for 3 months. Data are coming from clinical trials reported in the literature [12-16] where the Meaningful Effect is defined as a change of 2 scores from placebo in a three-month time-scale. The ADAS-Cog endpoint is the clinical outcome used in Alzheimer’s Disease trials.

The next example illustrates the value of re-using data from external sources. The golden standard measurement of efficacy for all approved Alzheimer’s Disease drugs, the ADAS-Cog endpoint, is visualized in Figure 7. The trellis plot visualizes results reported from different Donepezil clinical trials performed in different subpopulations [12-16] using Treatment Effect Index, where the Treatment Effect is given by an absolute change from placebo and the Meaningful Effect is a score of 2.0 in a 3-month study (supported by the clinically relevant level in Donepezil registered trials). Thus in that case, a TEI of 100 corresponds to a two-point improvement over placebo in the ADAS-Cog endpoint. The graph clearly demonstrates that the ADAS-Cog score shows different responsiveness in different subpopulations of Alzheimer’s Disease and that during the last years Donepezil had failed in several studies. These data were used to compare with one internal compound where Donepezil was used as a positive control (unpublished data), thus unbound concentration are used in the trellis plot. Using total concentration on the horizontal axis, will show the same pattern as subpopulations and compound are the same in all panels.

The value of real-time forward and back translation to enable informed decision has been recently been demonstrated [4]. Both retrospective and prospective integration of data resulted in improved cross-functional work and increased transparency of the large amount of compound and project information. Exploitation and interpretation of preclinical and clinical data supported improved awareness of face validity and predictability of animal models and in vitro assays. Retrospective documentation of all available data and information available at each milestone for decision-making clearly identified which preclinical and clinical studies the biomarkers and endpoints had reached a meaningful effect or a threshold. Another finding was hepatotoxicity in phase II, with no pre-warnings from preclinical data or early clinical studies, which clearly demonstrated that additional biomarkers are needed. The Knowledge Plot concept was also used to evaluate or benchmark compounds in due diligence (unpublished data). The prospective pilot initiative also highlighted the requirements on data and infrastructure for aligning a number of existing translational data platforms across pre-clinical and clinical domain. It also exposed the need to develop an enterprise solution in comparison to the methods used in the pilot projects, which were tailored to the individual project needs [4]. In addition to unbound and total concentration in plasma, drug exposures in CSF and brain were compared with efficacy biomarkers for a number of compounds and species in the prospective study (unpublished data).

The Knowledge Plot approach helps project teams to handle enormous amounts of information in a flexible way with limited efforts and without getting information overload. The Knowledge Plot visualizes and integrates available data and knowledge in a transparent way by enabling a holistic (cross domain) interpretation by taking all attributes into consideration. As of today we have about 15 different targets, 50 compounds, 10 species, 17 populations/subpopulations, 260 studies and 100 endpoints in our database and we continue to build the knowledge bank as new and existing compounds progress in the pipeline.

## Discussion

In this paper, the basic version of the Knowledge Plot has been presented to illustrate the main idea and how it can be implemented to support business needs. The concept can be, and has been, expanded in many different ways. Some characteristics that have been incorporated into the Knowledge Plot are: confidence in each observation (confidence intervals, standard error, and number of observations the summary is based on), individual data, and time dimension. Others have proposed a scoring system for biomarker assessment [17] and their translatability [18] in early drug projects to estimate risks in project and portfolio decisions. This scoring system apply weights on data in order to avoid weak data sets to have equal impact as strong data sets by using scores between 1 and 5 (e.g. 5 = more validated data, more clinically relevant data). This implies that therapeutic areas with high translational risk will already up front call for the need to identify more reliable biomarkers [19]. Such a weighting procedure can be a complement in the decision-making process.

The use of expert knowledge to define a desired Meaningful Effect, is the fundamental principle that distinguish the Knowledge Plot from methods published by others. One example is the utility function that is a component in the Clinical Utility Index (CUI) [8-10]. The CUI is a weighted sum of the utilities of all the attributes that are considered to be important for decision making in the actual situation. Both the CUI and each of the individual utilities take values in the (0, 1) range whereas the TEI can take any value though values in the (0,100) range are most common. In order to add a new summary index, in parallel to what is presented in this paper, you only need to convert it to the (0,100) scale. Clinical Utility Index can be used alternatively, or be included as complement, to the Treatment Effect Index.

There are some important considerations, in particular how an index such as the Treatment Effect Index is derived. One is that the random variation in the control group that is used to normalize against will result in even larger random variation in the normalized summary index, in particular when the control group is small in relation to the variation of the endpoint. Another concern is how to standardize the placebo/vehicle controlled treatment response. The Knowledge Plot uses standardization against the meaningful effect, which initially can be difficult to define for explorative endpoints. However, for such endpoints the relative difference between compounds can still be identified as long as the same meaningful effect value is used. Note that the reference value should be updated when more knowledge about the explorative endpoint is gained. Instead of using a reference value of a meaningful effect to derive an index it has been suggested to use the variance, or the standard deviation, to standardize the treatment effect to a normalized summary index, e.g. the Clinical Utility Index [8-10]. There are pros and cons with all methods. The method that suits the particular situation should be selected depending on how to interpret complex data and information with visualization techniques. The main reason why using the Treatment Effect Index is that it includes the expert opinion on the biological/medical knowledge identifying at which level an endpoint will give benefit to the patient. The index is based on current available treatments so that an index value of 100 will mean a biological meaningful treatment effect that has a realizable business value. A Napiergram can be derived by collapsing the vertical axis in the Knowledge Plot (see Figures 5 and 6, compound B).

Data standards and terminologies are very important in all data integration initiatives. Especially controlled vocabularies are important in order to compare across species, endpoints and studies. There are several available options that can be used (e.g. MeSH [20], Snowmed [21], MeDRA [11], NCI terminology [22]) as well as data structures and information models where the CDISC-suite [23] (SEND and SDTM) or a Triple Store solution may be considered. In general, the terminology used in clinical development is more consistent compared to the terminology in preclinical research.

Evaluating multiple attributes in a prospective manner and utilization of current information and knowledge are necessary procedures in order to optimize the productivity in future drug development. Visualization, data mining and knowledge management are all critical capabilities in this process. Currently, several stand-alone computational tools are used in a manual environment and data often exists in disconnected databases. Integration of data is a complex and difficult endeavor. Thus, a common computational infrastructure will remove many of the inherent road blocks [24]. In addition, cultural changes have to take place to ensure effective sharing and integration of data between various functions, such as Drug metabolism and pharmacokinetics (DMPK), Pharmacology, Safety and Clinical, and external sources. The principles enlisted here is key to achieve shorter development timelines and will allow more time spent on building a Translational and Knowledge continuum that can support all drug development stages. Incorporation of scientific knowledge allows the organization to make informed and transparent investment decisions with respect to individual projects as well as the complete portfolio. Furthermore, using frameworks like the one presented in this paper serves like a common language for the spectra of subject matter experts that constitute a modern drug development team. Each subject matter expert is accountable for the interpretation within respective domain (i.e. Meaningful Effect), but that same person can also easily accept the data-driven interpretations from all the other domains, that includes subject matter expertise from other functions. Ultimately, we have seen that the Knowledge Plot catalyzes the build-up of confidence and trust among the team members, where the discussion have moved from one experiment at a time to what the experiment is worth in the context of all available information and knowledge. The organizational effects are difficult to quantify, but informed decision making and knowledge management are central for large research and developing organizations. In this context the Knowledge Plot plays a central role and will be of great value for everyone that embraces its principles.

## Conclusions

The Knowledge Plot allows a transparent head-to-head comparison of data across multiple domains. It harmonizes and simplifies the interpretation and enables scientific and informed decision-making in various stages of drug development. Furthermore, the Knowledge Plot visualizes the translational and knowledge continuum, which in turn will reduce the attrition rate and reduce costs of clinical development by spotting poor candidates early. It provides a quick overview of what has been done with a molecule and uncovers hidden patterns by comparing the molecule with previous similar molecules and how efficacy and safety profiles compares with potential competitors and comparators. Exhaustive information, holistic understanding and integration of all current knowledge are prerequisites for effective decision making. Thus, the Knowledge Plot is a valuable communication, decision-making and knowledge management tool that will result in an improved understanding of the competitive edge for a particular project or disease area portfolio.

## Competing interests

The authors declare that they have no competing interests.

## Authors’ contributions

LB and NS initiated and developed the Knowledge Plot concept. AB built the infrastructure and information model. LB led the design of all visualizations and AB technically built all visualizations. LB, NS and AB drafted the manuscript. All authors read and proofed the final manuscript.

## Supplementary Material

Additional file 1: Table S1 Variables used in a basic Knowledge Plot. Click here for file
