# Supplementary material for: Effective visualization of integrated knowledge and data to enable informed decisions in drug development and translational medicine
Source: J Transl Med. 2013 Oct 8;11:250. doi: 10.1186/1479-5876-11-250 (PMC3842641; doi:10.1186/1479-5876-11-250)
Supplement: Additional file 1: Table S1 — Variables used in a basic Knowledge Plot. [file 1479-5876-11-250-S1.docx]

## Additional file 1

Table S1 Variables used in a basic Knowledge Plot

| Column | Description | Mandatory | Valid values |
| --- | --- | --- | --- |
| Species | Common term for species. Same name as in spreadsheet “*critical values”* | Yes | e.g. Human, Rat |
| **SpeciesDetail** | Exact term for species | No | e.g. Wistar rat, Sprague-Dawley rat |
| **Substance** | Name of the substance | Yes |  |
| **StudyType** | Common name for study type | No | e.g. Telemetry study, SAD study |
| **StudyCode** | Study code | Yes | e.g. AZ12345678910 |
| **Treatment** | Treatment group | Yes | e.g. 0.31 µmol/kg |
| **Ngroup** | Number of subjects per treatment group | No | e.g. 6, 11 |
| **ReportDate** | Date when results available, i.e. the date of publication | No | e.g. yyyy-mm-dd |
| **MilestoneAvailable** | Milestone when information were available, possible: pre MS4, post MS4 but pre MS5, and post MS5 | No |  |
| **Concentration** | Mean Cmax/Css/Cmin (during study time) in treatment group (may be derived from satellite groups). The same unit has to be used in the entire project, please convert concentrations if necessarily. | Yes | e.g. 0, 41.3 |
| **ConcCalc** | Description of how the concentration has been calculated | No | e.g. Concentration from satellite animals, Mean Cmax in treatment group |
| **FractionUnbound** | Unbound fraction for the drug in the actual species. Set this to 1 if you enter in vitro functional/binding assay (e.g. EC50, Ki och Kd values) | Yes (=1 for in vitro assays) | e.g. 0.097 |
| **ConcentrationUnit** | Concentration unit. The same unit has to be used for all studies, convert if necessary. | Yes | e.g. nmol/L |
| **Type** | Type of endpoint | Yes | Efficacy or Safety |
| **Endpoint** | Short name of the endpoint. Same name as in spreadsheet “critical values” | Yes | e.g. PWL(s), QTcF |
| **EndpointDetails** | Full description of the endpoint with unit in brackets | No | e.g. Diastolic arterial blood pressure (mmHg), Heart rate (bpm) |
| **EndpointUnit** | Unit of the endpoint | No | g |
| **Endpoint Value** | Mean/max/min value in treatment group (over a time period or for a particular time point depending on study). Use max/min for safety variables (the most extreme) and average (e.g. mean or AUC) for efficacy variables. | Yes (but not for NOAEL, LOAEL, EC50, Ki, or Kd etc.) | e.g. 32.2, 159 |
| **Comments** | Comments | No | Free text |
| *AEIntensity** | AE intensity | Only for clinical studies | Mild, Moderate, Severe |
| **AEClass*** | Organ Class Name or Preferred Term Name for visualization purposes | No |  |
| **AEOrganClass*** | MedDRA System Organ Class Name | Only for clinical studies | e.g. Cardiac disorders, Nervous system disorders |
| **AEPreferredTerm*** | MedDRA Preferred Term Name | Only for clinical studies | e.g. Dizziness |
